# Co-culture of *Helicobacter pylori* with oral microorganisms in human saliva

**DOI:** 10.1007/s00784-025-06160-4

**Published:** 2025-01-24

**Authors:** Konstantin Johannes Scholz, Annabelle Höhne, Annette Wittmer, Georg Häcker, Elmar Hellwig, Fabian Cieplik, Barbara Waidner, Ali Al-Ahmad

**Affiliations:** 1 Department of Operative Dentistry and Periodontology, Center for Dental Medicine, Medical Center– University of Freiburg, Faculty of Medicine, University of Freiburg, University of Freiburg, Freiburg, Germany; 2 Department of Conservative Dentistry and Periodontology, University Hospital Regensburg, Regensburg, Germany; 3 Institute of Medical Microbiology and Hygiene, Faculty of Medicine, Medical Center-University of Freiburg, University of Freiburg, Freiburg, Germany; 4 Department of Biochemistry and Chemistry, Philipps University of Marburg, Marburg, Germany

**Keywords:** *Helicobacter pylori*, Human saliva, Oral microorganisms, Co-culture experiments, Microbial Survival; oral microbiome; culture technique

## Abstract

**Objective:**

*Helicobacter pylori* is known for colonizing the gastric mucosa and instigating severe upper gastrointestinal diseases such as gastritis, gastroduodenal ulcers, and gastric cancer. To date, there is no data available on the oral cavity as transmission site, whether *H. pylori* can survive in the oral cavity or in human saliva. The aim of the study was to investigate the influence of oral microorganisms and human saliva on the survival of *H. pylori* in human saliva.

**Methods:**

*H. pylori* strains KE, a motile derivate of type strain *H. pylori* 26695, and *H. pylori* SS1, a clinical isolate from a gastric biopsy, were grown in human pooled saliva (pooled from 4 healthy human donors, 0.22 μm filter-sterilized) or in BBF (Brucella browth formula; control) either as mono-cultures or in co-culture with *Streptococcus mutans*, *Streptococcus oralis*, *Actinomyces naeslundii*, *Lacticaseibacillus casei* and *Candida dubliniensis*. Bacterial survival of *H. pylori* and the oral microorganisms were investigated using colony forming units (CFU) assay and MALDI-TOF MS at baseline and after 24, 48 and 168 h.

**Results:**

In saliva, *H. pylori* KE demonstrated enhanced survival in co-culture with *S. mutans*, *A. naeslundii*, and *C. dubliniensis*, enduring for at least 48 h. In contrast, *L. casei* and *S. oralis* inhibited *H. pylori* KE in saliva. *H. pylori* KE could not be cultured after 168 h in saliva, neither in mono- nor co-culture. In contrast, *H. pylori* SS1 in saliva could be cultured after 168 h in co-culture with *S. mutans* and *C. dubliniensis*, but not in mono-culture. In BBF, *H. pylori* KE could be cultured after 168 h with *S. mutans*, *L. casei* and *C. dubliniensis*, and *H. pylori* SS1 with *L. casei* and *C. dubliniensis*, but not with *S. mutans*. Notably, the co-cultured microorganisms survived at high CFU numbers similar to those of the monocultures.

**Conclusion:**

The study suggests that *H. pylori* can transiently survive in human saliva and even with presence of certain oral microorganisms. However, it may not be a permanent resident of the oral microbiota. The co-survival with oral microorganisms emphasizes the necessity for studying the role of the oral microbiota in the infectious and transmission cycle of *H. pylori*.

## Introduction

Consequences of the infection with *Helicobacter pylori*, a Gram-negative, spiral-shaped bacterium that can be highly motile due to switchable flagellar motility and was first isolated in a biopsy from the gastric surface epithelium of patients with active chronic gastritis [1–3], are a major health concern worldwide. *H. pylori* is microaerobic and is able to turn into its coccoid form under aerobic conditions, which might persist in the environment [4, 5]. *H. pylori* has been shown to be associated with various gastrointestinal diseases, such as chronic gastritis, peptic ulcers, dyspepsia and gastric malignancies [6–10]. Furthermore, *H. pylori* infection was shown to be associated with other diseases such as autoimmune diseases, cardiovascular and cerebrovascular diseases and iron-deficiency anemia [6, 11]. Due to its association with gastric cancer, *H. pylori* was classified as human group 1 carcinogen by the World Health Organization (WHO) and International Agency for Research on Cancer (IARC) [12, 13]. The prevalence of *H. pylori* infections depends on the socio-economic and hygiene conditions in different geographical regions and varies from less than 30% in some industrialized countries to up to 80% in lower- and middle-income countries [8, 14, 15] resulting in an estimated global prevalence above 40% in the period between 2011 and 2022 [16]. Although the transmission routes of *H. pylori* are still not entirely clear, the oral-oral, faecal-oral and gastro-oral transmission routes are strongly favored in the literature [8, 14]. Considering these transmission routes and the fact that person-to-person transmission of *H. pylori* within family members has been emphasized for the development of eradication strategies [7, 14], the role of the oral cavity in the transmission pathway of *H. pylori* has been a focus research interest [17].

Furthermore, *H. pylori* has been detected in animals, for example old-world macaques, which are rather unlikely to be an important reservoir for human infection [18]. However, identification of *H. pylori* has also been described in animals near the human habitat such as horses, calves, pigs and commercially reared cats [18, 19]. *H. pylori* may be transmitted between species due to water contaminated by faeces or vomit and further enter the food chain [19]. In humans, a predominantly within-family transmission is assumed [20, 21]. *H. pylori* has a long-documented association with humans, exemplified by its detection in the 5,300-year-old South Tyrolean “Iceman” mummy [20, 21]. This profound historical association, believed to span over 50,000 years, establishes *H. pylori* as a highly dependable indicator for tracking both recent and ancient human population movements [20, 22].

Regarding the survival of *H. pylori* in the human oral cavity, there are contradictory reports in the literature. A recent review article regarding the colonization of the oral cavity by *H. pylori* showed that various heterogeneous methods were used to detect *H. pylori* in the oral cavity, including immunological, biochemical, molecular biological and culture techniques [17]. While the culture technique has not yet provided any evidence that *H. pylori* could be isolated from the oral cavity apart from one study that described the cultivation of *H. pylori* from samples of root canal systems of deciduous teeth, but not from the corresponding dental plaque samples [23], all other methods showed high detection rates [17, 24, 25]. Furthermore, most studies have neglected the risk of cross-reaction with species similar to *H. pylori* such as *Campylobacter* spp. and the risk of contamination of the oral cavity with *H. pylori* through gastroesophageal reflux. Additionally, false-positive results of *H. pylori* have been reported by using non-invasive methods such as urea breath test, PCR (polymerase chain reaction) and LAMP (Loop-Mediated Isothermal Amplification**)** [26–28], which emphasizes the fact that caution is required when interpreting positive results for *H. pylori* in the oral cavity obtained by non-invasive methods. Interestingly, in a very recent study and based on results of ELISA tests, which detect *H. pylori* antibody IgG in human saliva, conclusions were drawn that dentists and dental students are at higher risk to be infected with *H. pylori* than other population groups [29]. Based on their results, the authors of the aforementioned study recommended special attention to dentists and dental students when studying *H. pylori* epidemiology although *H. pylori* has not been cultivated from any of the saliva samples taken from the study subjects. Using nested PCR, another study revealed also a higher prevalence of *H. pylori* infection among dentists and recommended intensive attention to be paid to oral infection by *H. pylori*, especially due to the correlation of such infections with the frequency of clinical practice per week [30]. In contrary to the aforementioned two studies, Lin and colleagues reported based on ELISA detection of *H. pylori* antibody IgG in human blood samples of 195 dental professionals that dentists, dental nurses, fifth year dental students and first year dental students do not have a higher prevalence of *H. pylori* antibody in comparison to normal population controls [31].

The transmission of *H. pylori* via human saliva cannot be ruled out, assuming that this bacterium can survive in human saliva and in co-culture with other oral microorganisms. Although this point is of great importance for clarifying the transmission routes of *H. pylori* and for assessing the risk for dental personnel, the survival of this bacterium in human saliva without and with other typical oral microorganisms has not yet been investigated. Hence, the aim of the present study was to investigate the survival of a type strain and a clinical isolate *of H. pylori* in human saliva without and with different typical oral microorganisms.

## Materials and methods

### Human pooled saliva collection

Four healthy volunteers provided unstimulated human saliva (50 mL Falcon, Becton Dickinson Labware, Franklin Lakes, USA). The inclusion criteria for donors of the saliva were as follows: periodontal health (periodontal screening index ≤ 2), non-smoking status, absence of medication use (especially antibiotics) for the three months prior to the beginning of the study with the exception of oral contraceptives, and the absence of *H. pylori* detectability in both stool (antigen detection via ELISA, test kit RIDASCREEN^®^, FemtoLab, FA R-Biopharm-AG) and saliva samples (PCR, primer pair EHC-U/EHC-L; [32]). Saliva of all volunteers was pooled and centrifuged at 2058×*g* for 10 min. Following the removal of the supernatant, the saliva was sterile-filtered (0.22 μm, Millex Millipore, Merck, Darmstadt, Germany). Afterwards, the saliva was stored at -80 °C. The ethics commission of the University of Freiburg approved the collection of human saliva from the sample donors who gave their written consent (23-1537-S1-AV).

### Strains and culture conditions

Experiments were conducted with *H. pylori* KE 88-3887, a motile derivative of *H. pylori* 26695, herein after referred to as *H. pylori* KE. To verify results with this reference strain, experiments were repeated with a *H. pylori* isolate (*H. pylori* Sidney Strain 1), which was originally isolated from a gastric mucosa biopsy of an *H. pylori*-positive patient [33, 34].

The present study further included *Streptococcus mutans* (DSM 20523), *Streptococcus oralis* (ATCC 35037*)*, *Actinomyces naeslundii* (DSM 17233*)*, *Lacticaseibacillus casei* (DSM 20011*)*, and *Candida dubliniensis* (RV ST. C*)*.

Both *H. pylori* strains were grown on DENT selective agar plate under microaerophilic conditions (Anoxomat, MART/gemini, Laan, Netherland) for 2 days at 36 °C. The oral microorganisms were grown on Columbia Blood Agar (CoBl) plates with 5–10% CO_2_ (Heraeus, Hanau, Germany) at 36 °C for 2 days.

Subcultures of *H. pylori* and the respective oral microorganisms were then established in cell culture flasks (Cellstar Cell Culture Flasks, Greiner Bio-One GmbH, Frickenhausen, Germany) with 15 mL of Brucella Broth Formula liquid medium (BBF: Brucella boullion with 5% Fetal calf serum) and incubated for 24 h at 36 °C. The *H. pylori* subcultures were grown microaerophilically under gentle agitation at 36 °C. After 24 h, the optical density (OD) of the subcultures was measured using a spectrophotometer (Ultrospec 4000, UV/Visible Spectrophotometer, Pharmacia Biotech, Uppsala, Sweden) at 600 nm against a blank with BBF culture medium, yielding an OD of 1.0 for the experiments.

### Culture of H. pylori in BBF or saliva with or without other microorganisms

- BBF + 10 vol% *H. pylori*.
- BBF + 10 vol% oral microorganism.
- BBF + 5 vol% *H. pylori* + 5 vol% oral microorganism.
- Pooled saliva + 10 vol% *H. pylori*.
- Pooled saliva + 10 vol% oral microorganism.
- Sterile-filtered pooled saliva + 5 vol% *H. pylori* + 5 vol% oral microorganism.

All of these experimental groups were investigated for 4 different evaluation timepoints:

- T0: immediately.
- T1: 24 h.
- T2: 48 h.
- T3: 168 h (7 days).

For each of these experimental groups and timepoints, a separate series of glass tubes each containing 2 mL of the inoculated culture were prepared to avoid contamination during repeated opening. These glass tubes were sealed with a breathable sterile cellulose stopper (Herenz, Hamburg, Germany), and grown under microaerophilic conditions at 36 °C until the respective evaluation timepoints (T0, T1, T2, T3).

From the inoculated cultures in the glass tubes, 10 µL were microscopically examined for contamination at each timepoint, and then 0.1 mL of these cultures were used to prepare tenfold dilution series with Peptone-Yeast-Extract Boullion (10 g pancreatic peptone from casein, 5 g NaCl, 2.0 g beef extract; all Merck, Darmstadt, Germany; 5.0 g yeast extract; Difco, Franklin Lakes, USA; 0.3 g cysteine hydrochloride, SERVA Electrophoresis GmbH, Heidelberg, Germany; ad 1000 mL distilled water) to determine colony-forming units per ml (CFU/mL) on agar plates. To minimize exposure of *H. pylori* to room atmosphere, it was ensured that liquid cultures were removed from the microaerophilic environment not longer than 45 min. Each dilution step was plated both on DENT and CoBl agar plates in duplicates. DENT plates were incubated under microaerophilic conditions for 5 days at 36 °C, CoBl plates were incubated for 2 days at 36 °C in a CO_2_ incubator. All experiments were repeated at least 6 times. At each timepoint, two plates were inoculated and the respective median was calculated. All strains were confirmed using MALDI-TOF MS as described in detail by Anderson et al. [35].

### Data analysis

CFU data were depicted as medians and neighboring quartiles (25% and 75% percentiles) from the results of six independent experiments at least. The figures were created using GraphPad Prism (v. 10, GraphPad Software, Boston, Massachusetts, USA).

## Results

**Fig. 1:**
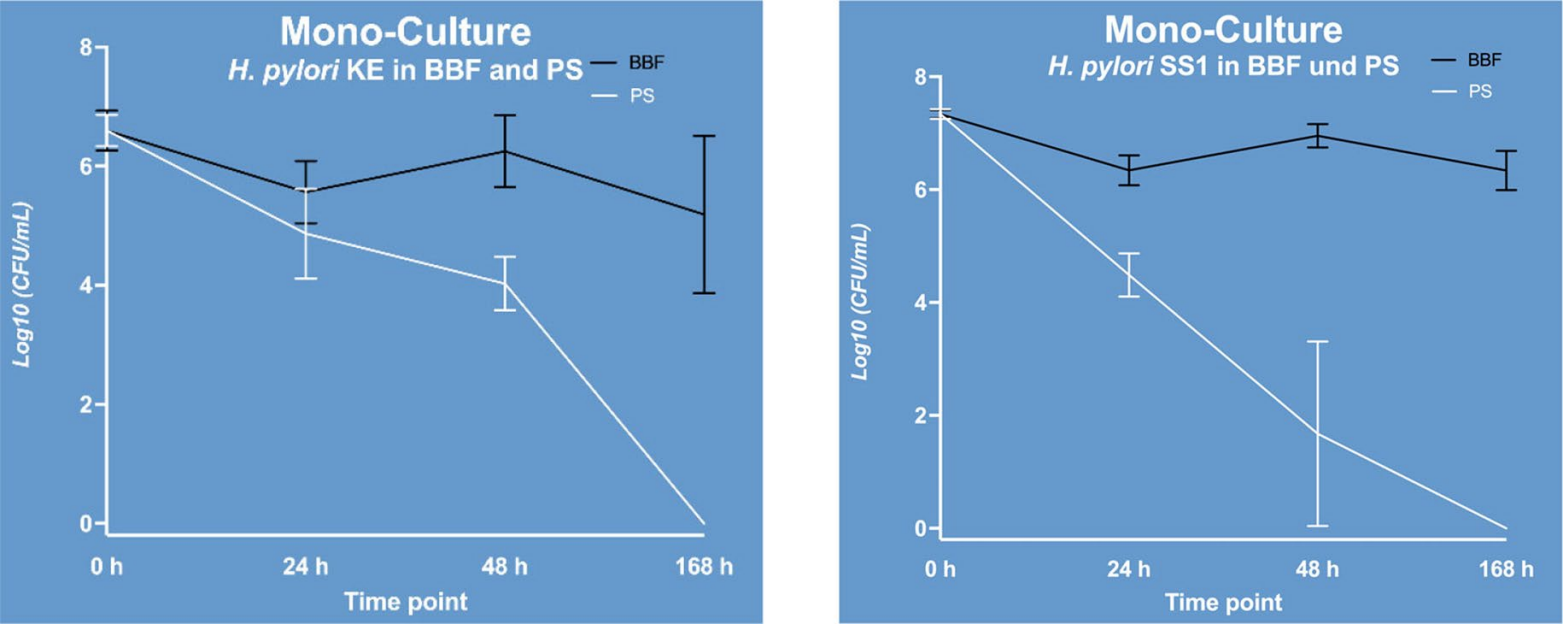
Log_10_ Colony forming units per milliliter (CFU/mL) for *H. pylori* KE and SS1 monocultures in Brucella Broth Formula (BBF) or Pooled Human Saliva (PS)

Bacterial counts of *H. pylori* KE and *H. pylori* SS1 in BBF monoculture remained stable over a week with fluctuations of 1–2 log steps (Fig. 1). To demonstrate no reduction of bacterial ability to replicate over this period was the basic prerequisite for the investigation of the selected *H. pylori* strains. In pooled saliva, a more pronounced decrease in CFUs was observed until no culturable bacteria were detectable at 168 h (Fig. 1).

**Fig. 2:**
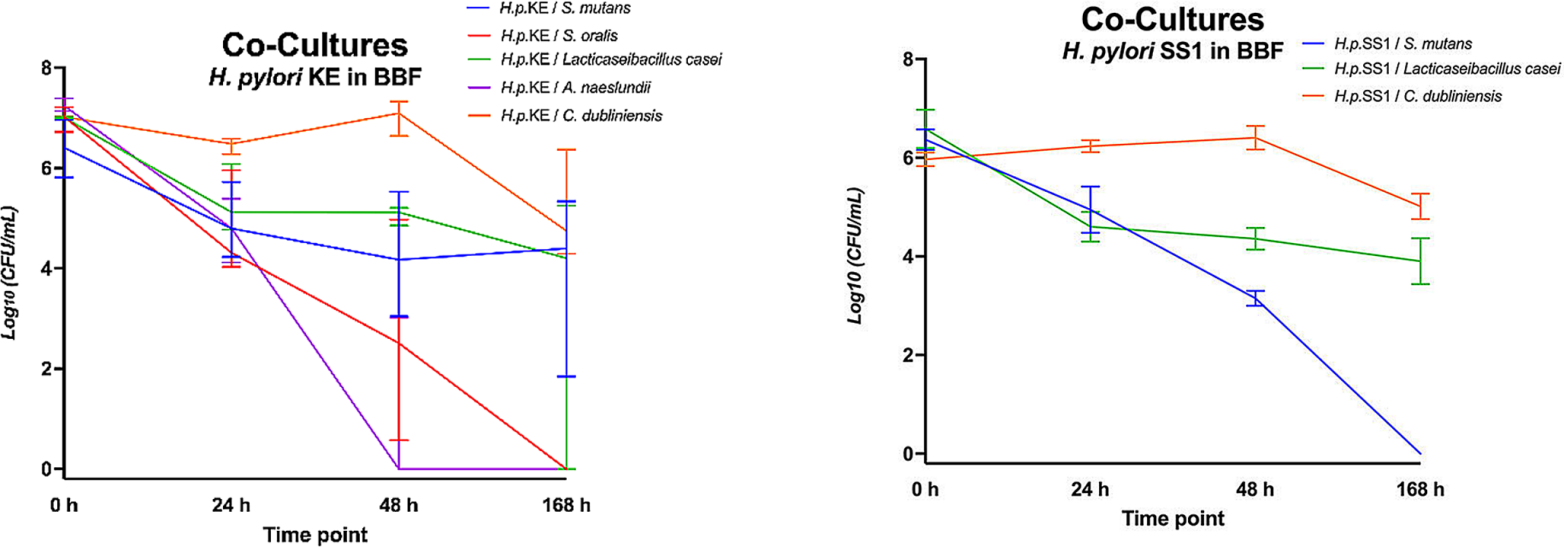
Log_10_ Colony forming units per milliliter (CFU/mL) for co-cultures in Brucella Broth Formula (BBF) *of H. pylori* KE combined with *S. mutans*, *S. oralis*, *L. casei*, *A. naeslundii*, *C. dubliniensis* and SS1 combined with *S. mutans*, *L. casei* or *C. dubliniensis*, respectively.

In co-culture in BBF (Fig. 2), the growth of both *H. pylori* KE and *H. pylori* SS1 was strongly influenced by the oral microorganisms. *H. pylori* KE was still detectable with > 10^4^ CFU/mL in median after 168 h co-culture with *S. mutans*, *L. casei* and *C. dubliniensis*. With the oral microorganism *S. oralis*,* H. pylori* KE could still be detected with > 10^2^ CFU/mL after 48 h, but not after 168 h. In co-culture with *A. naeslundii*, *H. pylori* KE was no longer detectable after 48 h. *H. pylori* SS1 in BBF was detectable with > 10^4^ CFU/mL at all timepoints in co-culture with *L. casei* and *C. dubliniensis*. Co-cultured with *S. mutans*, it revealed > 10^3^ CFU/mL at 48 h but no detectable CFU after 168 h.

**Fig. 3:**
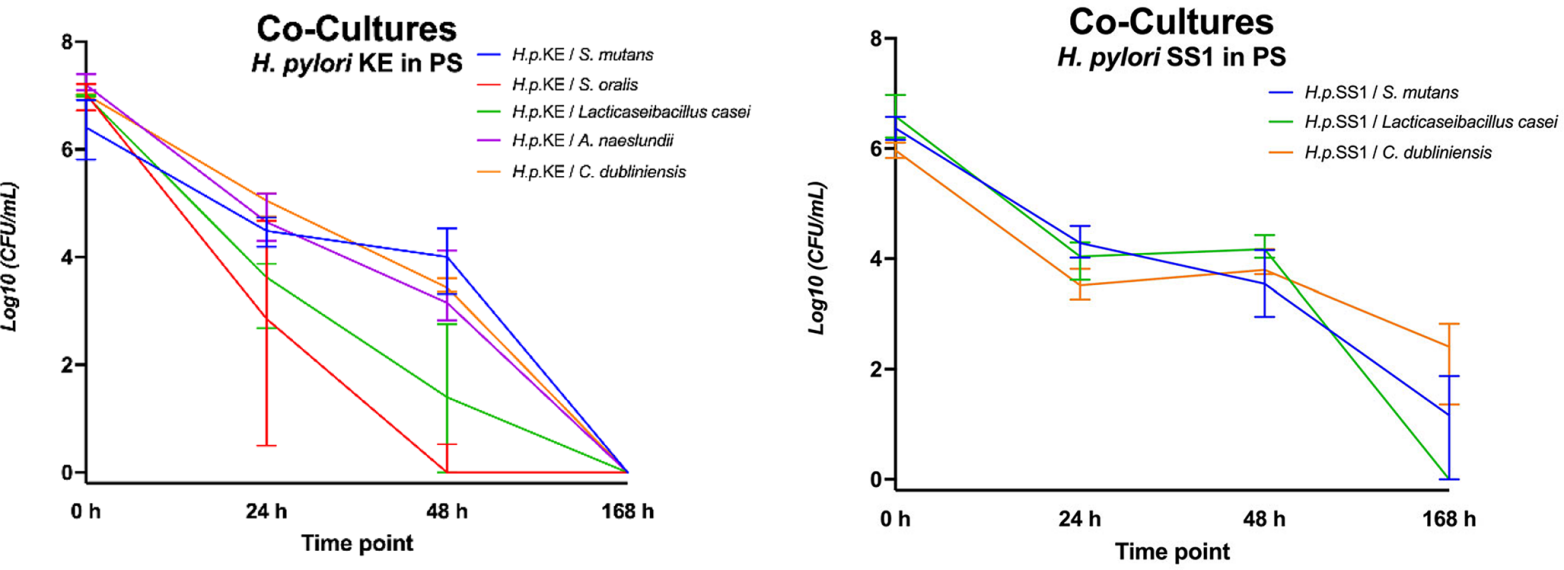
Log_10_ Colony forming units per milliliter (CFU/ml) for co-cultures in human pooled saliva (PS) *of H. pylori* KE combined with *S. mutans*, *S. oralis*, *L. casei*, *A. naeslundii*, *C. dubliniensis* and SS1 combined with *S. mutans*, *L. casei* or *C. dubliniensis*, respectively

**Fig. 4:**
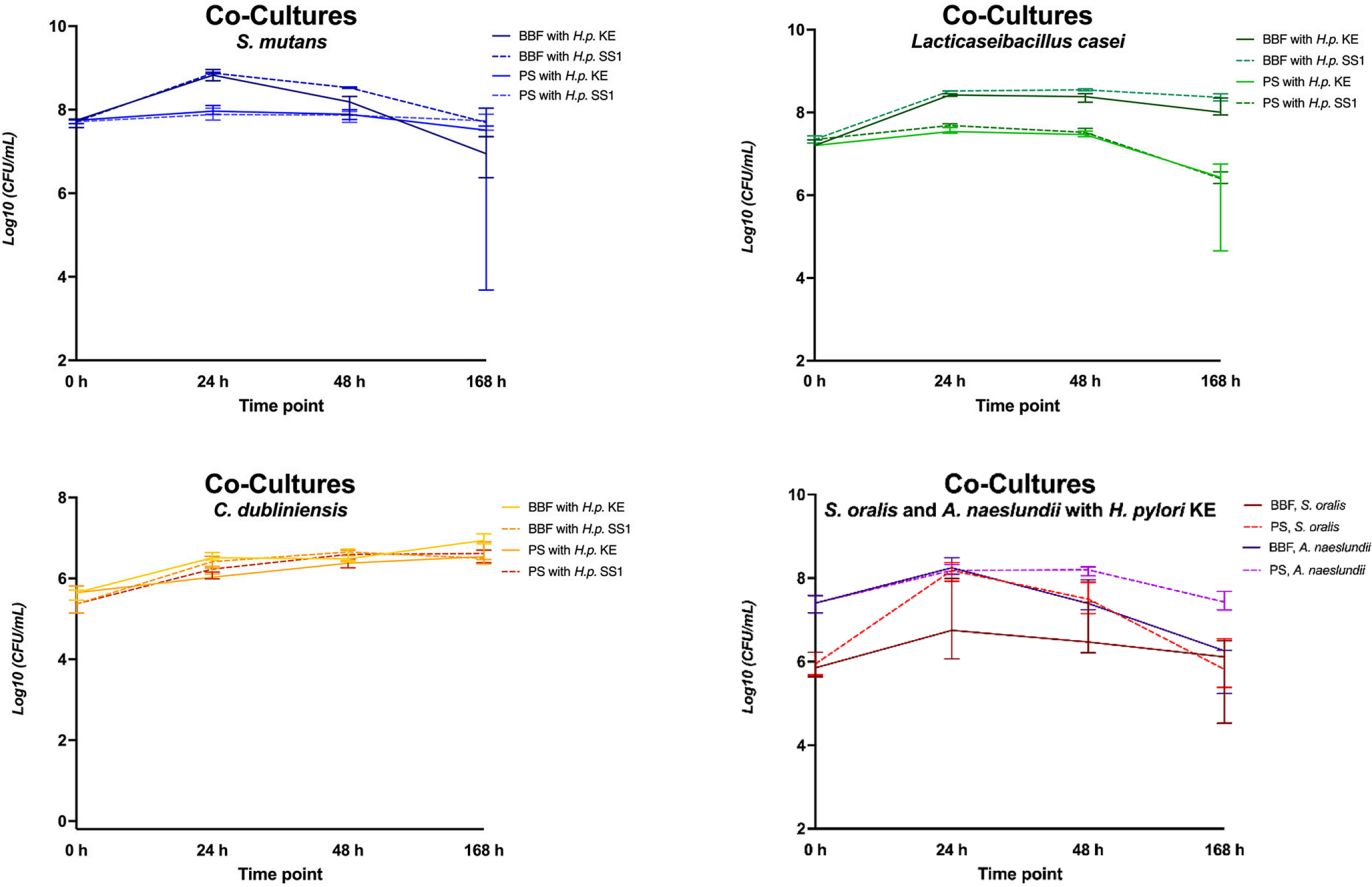
Log_10_ Colony forming units per milliliter (CFU/mL) for respective oral microorganisms from experiments shown in Figs. 2 and 3 in human pooled saliva (PS) or Brucella Broth Formula (BBF) of* H. pylori* KE, *S. oralis* and *A. naeslundii* were investigated in co-culture with* H. pylori* KE, whereas *S. mutans*, *L. casei* and *C. dubliniensis* were investigated in co-culture in separate experiments with both, *H. pylori* KE and SS1, respectively. All oral microorganisms revealed stable growth over the whole experimental period of 168 h

In human pooled saliva (PS; Fig. 3), *H. pylori* KE demonstrated growth in co-culture with *S. mutans*, *A. naeslundii*, and *C. dubliniensis*, enduring for at least 48 h with > 10^3^ CFU/mL. In contrast, *L. casei* showed a slightly inhibitory effect on CFU of *H. pylori*. *S. oralis* inhibited *H. pylori* KE in terms of no CFU detectable after 48 h and 168 h. *H. pylori* KE could not be cultured at all after 168 h in pooled saliva, either in mono- or co-culture. CFU were detectable for *H. pylori* SS1 after 168 h in co-culture with *S. mutans* and *C. dubliniensis*, but not in mono-culture. The co-cultured oral microorganisms survived at high CFU numbers at all investigated timepoints similarly to their mono-cultures (Fig. 4). *H. pylori* KE showed superior survival co-cultured with *A. naeslundii* in human pooled saliva compared to BBF.

## Discussion

Although *H. pylori* has been the subject of intense research over the past four decades, the pathway of transmission and the role of the oral cavity in it have not been fully elucidated. Therefore, the present study focused on the survival of *H. pylori* in human saliva with and without typical oral microorganisms. We were able to show that both, a *H. pylori* KE strain as well as a clinical isolate (SS1), were able to survive after 48 h of incubation in human pooled saliva with and without co-incubation with typical oral microorganisms. Furthermore, the clinical isolate SS1 was culturable after 168 h of co-culture with *S. mutans* or *C. dubliniensis* in human pooled saliva.

To the authors’ best knowledge, the survival of *H. pylori* in unstimulated human saliva for up to one week with or without typical oral microorganisms has not been reported in the literature. In particular, culture techniques have rarely been used to study the survival of *H. pylori* in human saliva or the oral cavity [17, 24, 25]. In the present study, both *H. pylori* strains showed clearly detectable growth and a high survival rate after one week of incubation in the Brucella Broth Formula culture medium, which emphasizes the suitability of this medium for growth control. Similar results for different *H. pylori* strains were shown in a previous study by Sainsus et al. [36]. Furthermore, the BBF medium enabled growth of *H. pylori* for up to one week after co-incubation with *S. mutans*, *L. casei* or *C. dubliniensis.* However, specific and optimized culture media to test the survival of *H. pylori* with oral bacteria may not be sufficient to draw conclusions about the real situation in the oral cavity. Accordingly, when using pooled unstimulated human saliva as culture medium, only the clinical isolate was culturable and only in low bacterial counts after one week of co-incubation with *S. mutans* and *C. dubliniensis*. The question arises whether, in addition to the coexistence *of H. pylori* and oral bacteria shown in the present study, there may be also symbiotic, epibiotic, or nursing microbes in the oral cavity that support or enable the growth, persistence, and transmission of *H. pylori*. In the past, it was discussed whether *C. dubliniensis* could even allow intracellular survival of *H. pylori* [37]. However, this is contradicted by the fungus’ stable cell wall, and there is also no evidence for this in the present study [37].

One limitation of the present study is that the bacteria were investigated in planktonic co-cultures and not in biofilm models. In the oral cavity, the biofilm, which can typically cover tooth and restoration surfaces and in which bacteria are also present in the form of organized, biofilm-like loose flakes, could on the one hand be a reservoir for a longer presence of *H pylori* [38, 39]. On the other hand, however, a stable and commensal biofilm with high diversity could also be unfavorable for permanent incorporation of new pathogens [40]. Furthermore, the oral cavity is characterized by food intake, circadian differences in saliva composition and secretion volume and an environment with changing conditions in terms of oxygen supply, pH-value, buffer capacity of these pH-fluctuations and temperature [41]. In the stomach, there are also fluctuations in pH-values, especially in different anatomic areas, but these tend to be in a more acidic range below 4, which makes the presence of competing bacteria for *H. pylori* less likely [42, 43]. These parameters could not be reproduced by the present study, but in particular the fluctuations in pH-value that occur during food intake could be an interesting topic for future studies.

The lower replication ability found for both *H. pylori* strains in human pooled saliva in general could be due to the antimicrobial salivary ingredients such as lysozymes, lactoferrin, statherin, lactoperoxidase, mucins, histatins, and immunoglobulin A [44, 45]. Nevertheless, the present study showed that the choice of bacterial strains has an important impact on the survival of *H. pylori* in saliva, as only with co-incubation with *S. mutans* or *C. dubliniensis*, the clinical isolate was detectable for up to one week in human saliva. Intriguingly, co-culture with *S. mutans* or *C. dubliniensis* seemed to facilitate the survival of *H. pylori* SS1 in human pooled saliva compared to the respective *H. pylori* mono-culture, which partly contradicts to a study of Ishihara et al., which stated that oral bacteria might inhibit *H. pylori* growth by producing bacteriocin-like inhibitory proteins against *H. pylori* strains [46]. The differences between both *H. pylori* strains in the present study may be related to genetic heterogeneity of clinical isolates compared to type strains. Such genetic heterogeneity could, for example, be due to natural competence leading to *H. pylori* strains with increased chronic infection capability [47]. The fact that the origin of bacteria plays a major role in their susceptibility to antimicrobial agents such as antibiotics has been demonstrated for various bacteria such as *Pseudomonas aeruginosa*, *Enterobacter* spp. and *Acinetobacter baumannii* [48–50]. In all the studies mentioned, clinical isolates were more found more resistant to antibiotics [48–50]. This again emphasizes the need to test clinical isolates of *H. pylori* in order to draw conclusions about its survival in human saliva and in the oral cavity.

Studies including molecular methods such as PCR coupled with an envious specificity of detection can lead to false positive results for *H. pylori* and to an overestimation of the survival of this pathogen in the oral cavity. For these reasons, the culture technique should always be used in parallel with PCR-based methods to confirm positive results for the presence of *H. pylori* in the oral cavity. Only when employing culture techniques, it can be assumed that *H. pylori* does indeed not only survive transiently in the oral cavity, but also could become resident there under certain conditions [17, 25].

The results of the present study should be confirmed by analyzing the survival of *H. pylori* together with the entirety of salivary bacteria in unstimulated saliva. Additionally, the survival of *H. pylori* after incubation with supra- and subgingival samples should be investigated to evaluate influences of possible co-aggregation with other oral microorganisms [39]. However, the supragingival and subgingival microbial composition strongly depends on oral hygiene and periodontal health, but existing publications have not identified any associations between *H. pylori* detection and periodontal parameters or poor oral health [51, 52]. However, there might also be some interindividual different factors in the oral cavity, such as low levels of certain chemorepellents or the presence of coccoid-stimulating factors, facilitating the persistent colonization of *H. pylori* in the oral cavity [53].

Another interesting point to investigate is whether contact with human saliva, which may act as a stressor for *H. pylori*, can induce a state of dormancy or the *H. pylori* coccoid form (HPCF), which is known as viable but non-culturable (VBNC) state [54–56]. Such a state is considered as survival strategy and generally gives the bacteria greater tolerance to environmental influences such as antimicrobial substances [57]. There is evidence that *H. pylori* has the potential to transform into a coccoid, unculturable state [58]. This would be of great importance for survival in the oral cavity and should be investigated in future studies.

## Conclusion

*H. pylori* can transiently survive in human saliva, even with and in some cases favored by the presence of certain oral microorganisms. However, there is still a lack of clear evidence if it can be a permanent inhabitant of the oral cavity. It is crucial for future research to study the role of the oral cavity and the resident microbiome in the infectious cycle of *H. pylori*.

## Data Availability

No datasets were generated or analysed during the current study.
